# Charing Cross Hospital

**Published:** 1897-11-13

**Authors:** 


### CHARING CROSS HOSPITAL.

A meeting of the Special Appeal Committee of the Charing Cross Hospital was held on Monday, the 8th inst., at the Mansion House. Lord Glenesk presided, and amongst those present were: Sir Joseph Fayrer, Bart., K.C.S.I., Sir Stuart Knill, Bart., Lieut.-Colonel Clifford Probyn, the Rev. Prebendary Kitto, Dr. J. Watt Black, Dr. Amand Routh, Mr. J. Astley Bloxam, Mr. John H. Morgan, Mr. A. G. Sandeman, and Mr. J. Hampton Hale.

The Chairman, after briefly giving a history of the appeal, said he thought the fact that, instead of only collecting one-fifth of the £100,000 appealed for, a sum of £35,000 had been raised in this, the first, year, was a most happy augury for the ultimate success of the appeal, especially when the special circumstances of the past year were taken into consideration.

A report from the committee was read by the Rev. Prebendary Kitto, in which it was stated that the £100,000 which they were trying to raise during the next five years was needed for the following objects: £50,000 for maintenance (*i.e.*, to pay off existing liabilities of over £17,000, and to meet the estimated deficiency of income during the five years), and £50,000 for new buildings (to include a new out-patients' department, a nursing home, special wards for special cases, and rooms for the resident medical staff). Altogether £34,627 had been collected to date, out of which the debt of £17,471 had been paid, and the freehold of the remaining portions of the hospital had been acquired.

On the motion of Mr. J. Hampton Hale, seconded by Mr. A. G. Sandeman, the report was adopted.

Mr. John H. Morgan, in proposing the following resolution:—

"That this meeting gratefully acknowledges the liberal support which the special appeal has received, and pledges itself to do all in its power to raise the sum still needed to render Charing Cross Hospital duly efficient for the great work which it carries on,"

said that the hospital was either in possession of, or negotiations were now pending for the purchase of the freehold of Toole's Theatre and of two houses in Chandos Street and King William Street. When these buildings were used for the purposes of the hospital, however, there would be a loss of about £1,500 a year—the net rents hitherto received by the hospital for these premises; besides which a good deal of capital had been sunk in the purchase of these freeholds He wished the public to understand that there was no idea of increasing the size of Charing Cross Hospital. The only object the committee had in view was to render the hospital better able to carry out the great objects which it had always endeavoured to carry out under the most disadvantageous circumstances.

The Rev. Prebendary Kitto seconded, and Sir Stuart Knill, Bart., supported the resolution, which was carried.

A vote of thanks to Lord Glenesk for presiding, and to the Lord Mayor for the use of the Mansion House, which was proposed by Sir Joseph Fayrer, Bart., seconded by Lieut.-Colonel Clifford Probyn, terminated the proceedings.

